# SLPI: a new target for stopping metastasis

**DOI:** 10.18632/aging.101372

**Published:** 2018-01-20

**Authors:** Lance L. Munn, Igor Garkavtsev

**Affiliations:** 1 Department of Radiation Oncology, Massachusetts General Hospital, Harvard Medical School, Boston, MA02114, USA

**Keywords:** SLPI, FoxM1, anti-metastatic compound

Secretory leukocyte protease inhibitor **(**SLPI) is known to be involved in the inhibition of inflammation, the modulation of immunological responses and the promotion of cell proliferation [1]. In cancer, increased SLPI expression can be detected in breast, lung, gastric and colorectal carcinomas. Using antibody arrays that identify a panel of 350 murine and 500 human secreted proteins, we screened cancer cells to identify proteins that are highly expressed in metastatic compared to their non-metastatic counterparts. We found that SPLI was the only secreted protein increased in metastatic variants of both breast and colorectal cancer cells. Moreover, the SLPI level was associated with increased lung metastasis from orthotopically implanted tumors. These results suggest that high expression of SLPI in cancer cells may promote lung metastasis [2].

Next, we checked how SLPI expression levels correlate with the incidence of triple negative breast cancer (TNBC) metastasis in patients. We analyzed SLPI expression in TNBC because this subgroup represents the most aggressive breast cancer and still has the worst prognosis, despite advancements in modern therapeutics. We found that both distant metastasis-free survival and overall survival were significantly worse in the TNBC patients who had higher SLPI expression in their tumors.

If SLPI is involved in tumor growth and metastasis, then pharmacological repression of SLPI should inhibit tumor expansion and dissemination. To identify potential small molecule blockers of SLPI, we used high-throughput screening to probe a library comprised of 60,000 chemically-diverse compounds. Candidate molecules that were able to reduce the SLPI expression by more than 90% but were not toxic to non-malignant cells were then tested in our mouse model of metastasis. We found that pharmacological repression of SLPI slows the growth of primary tumors and inhibits their dissemination to the lung [2]. These results suggest that SLPI is a new target for anti-metastatic therapies for breast and colon cancers.

**Figure 1:**
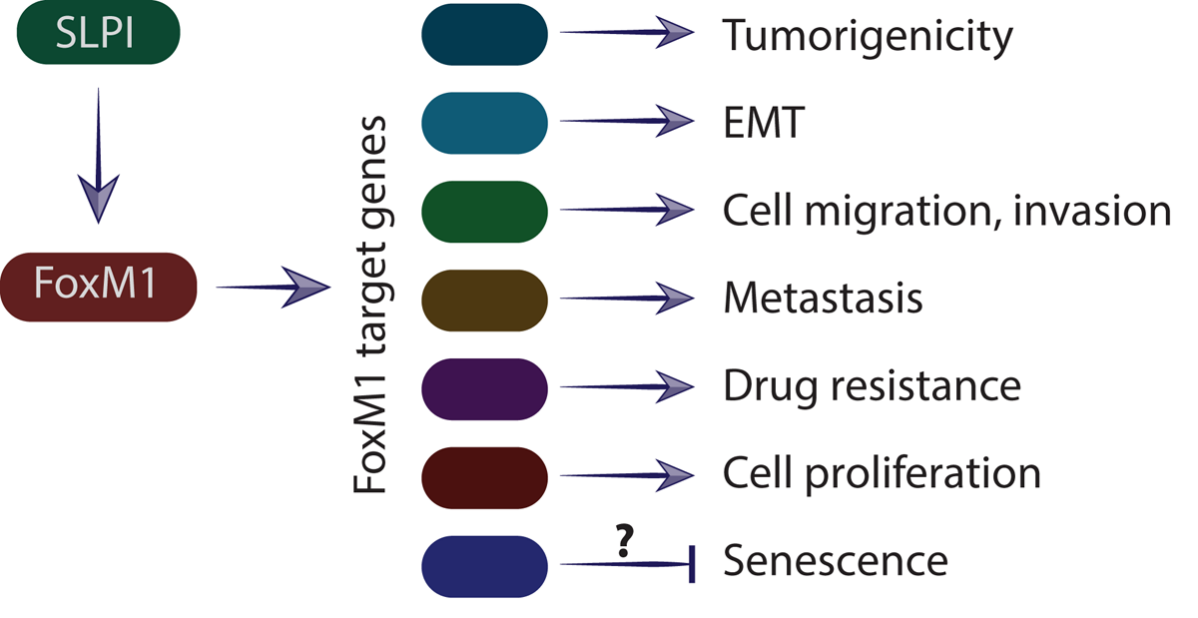
**Schematic representation of SLPI effect on the regulation of the FoxM1 target genes and subsequent metastasis.** SLPI activates transcriptional factor FoxM1, which stimulates expression of target genes involved in cell division, cell migration, premetastatic niche formation, epithelial–mesenchymal transition, senescence and metastasis.

What is a possible mechanism by which SLPI promotes metastasis? First, we found that SLPI physically interacts with and phosphorylates retinoblastoma (Rb) tumor suppressor protein. To determine whether SLPI interacts with Rb, we used protein extracts from two different cell lines and precipitated Rb complexes for SLPI detection. Our experiments confirmed that there is a physical association between these two proteins. This interaction was further confirmed by the reverse experiment in which anti-SLPI antibody was used for immunoprecipitation and anti-Rb antibody was used for detection. For further verification of this interaction, we used a standard mammalian two-hybrid approach and our results indicate that SLPI protein directly physically interacted with the Rb tumor suppressor. Moreover, we found that treatment with recombinant SLPI protein increased phosphorylation of Rb. On the other hand, treatment with the anti-SLPI compound decreased phosphorylation of Rb in breast cancer cells. Interestingly, it is known that phosphorylation of Rb leads to its release from FoxM1 binding [3] and initiates FoxM1 transcriptional activity. Indeed, we found that overexpression of SLPI leads to increased levels of known FoxM1 target proteins, such as cyclin B1, aurora kinase B and superoxide dismutase-2 (SOD-2). Moreover, we found that SLPI increases FoxM1 binding to FoxM1 target genes (Figure 1).

Could SLPI overexpression cause cellular senescence through activation of the FoxM1? There is a body of literature showing that FoxM1-depletion is sufficient to induce cellular senescence in proliferating cells [4]. Activation of the FoxM1 gene suppresses reactive oxygen species (ROS) and protects cancer cells from senescence [5]. Thus, overexpression of SLPI might prevent cellular senescence in cancer cells. On the other hand, FoxM1also regulates c-myc expression which has been shown to activate cellular senescence. Therefore, the effects of SLPI on cellular senescence warrants further investigation.
